# Lung cancer surgery after COVID-19 infection in a patient with severe interstitial pneumonia and restrictive ventilatory impairment

**DOI:** 10.1186/s40792-022-01531-5

**Published:** 2022-09-21

**Authors:** Hiroaki Komatsu, Nobuhiro Izumi, Takuma Tsukioka, Hidetoshi Inoue, Ryuichi Ito, Satoshi Suzuki, Noritoshi Nishiyama

**Affiliations:** Department of Thoracic Surgery, Osaka Metropolitan University Hospital, 1-4-3 Asahi-Machi, Abeno-Ku, Osaka, 545-8585 Japan

**Keywords:** Lung cancer, Surgery, COVID-19, Interstitial pneumonia, Restrictive ventilatory impairment

## Abstract

**Background:**

The spread of COVID-19 infection increased the number of patients who underwent pulmonary resection for lung cancer after COVID-19 infection. It is unclear how previous infection with COVID-19 affects perioperative complications and acute exacerbation of interstitial pneumonia after surgery in patients with interstitial pneumonia.

**Case presentation:**

An 80-year-old man was referred to our hospital because of a tumor in his left lung. Chest computed tomography showed a 28-mm nodule in the lower lobe of the left lung and usual interstitial pneumonia in bilateral lungs. Bronchoscopic examination was performed, which diagnosed squamous cell carcinoma. Pulmonary function testing revealed restrictive ventilatory impairment, and we planned to perform basal segmentectomy of the left lung. However, before the planned surgery, the patient contracted symptomatic COVID-19. Chest computed tomography revealed ground-glass opacities owing to COVID-19. The patient was admitted for surgery 7 weeks after COVID-19 infection. Preoperatively, pulmonary function testing was repeated, which revealed decreased % vital capacity (%VC) and % diffusing capacity for carbon monoxide (%DLco). The 6-min walk test indicated a distance of 500 m, and the percutaneous oxygen saturation at the end of the test was 94%. Basal segmentectomy of the left lung was performed by video-assisted thoracoscopic surgery. The patient’s postoperative course was favorable, and he was discharged without the need for oxygen inhalational therapy 12 days after the surgery. Pathological examination of the resected specimen revealed usual interstitial pneumonia in the non-cancerous areas of the lung. Additionally, the infiltration of immature fibroblasts in the alveoli and perivascular infiltration of inflammatory cells were observed, which were consistent with fibrotic change after inflammation owing to COVID-19. Three months after the surgery, the patient was alive without recurrence or acute exacerbation of the interstitial pneumonia. Pulmonary function testing 6 weeks after surgery revealed decreased %VC and %DLco. Testing 12 weeks after surgery revealed persistently decreased %VC and improved %DLco (Table 1).

**Table 1:** Pulmonary function test results before and after COVID-19 infection and 6 and 12 weeks after surgery

	VC (ml)	%VC (%)	%DLco (%)
Before COVID-19 infection	2070	71.9	74.9
7 weeks after COVID-19 infection	1700	59.6	51.9
6 weeks after surgery	1500	52.6	53.1
12 weeks after surgery	1510	53.0	61.7

*%VC* % vital capacity, *%DLco* % diffusing capacity for carbon monoxide

**Conclusion:**

We successfully performed basal segmentectomy of the left lung for lung cancer 7 weeks after COVID-19 infection in a patient with severe interstitial pneumonia and restrictive ventilatory impairment.

## Background

The spread of COVID-19 infection increased the number of patients who underwent pulmonary resection for lung cancer after COVID-19 infection [1–4]. It is unclear how previous infection with COVID-19 affects perioperative complications and acute exacerbation of interstitial pneumonia after surgery in patients with interstitial pneumonia. We herein report a patient with severe interstitial pneumonia and restrictive ventilatory impairment who underwent basal segmentectomy of the left lung for lung cancer 7 weeks after COVID-19 infection.

## Case presentation

**Fig. 1:**
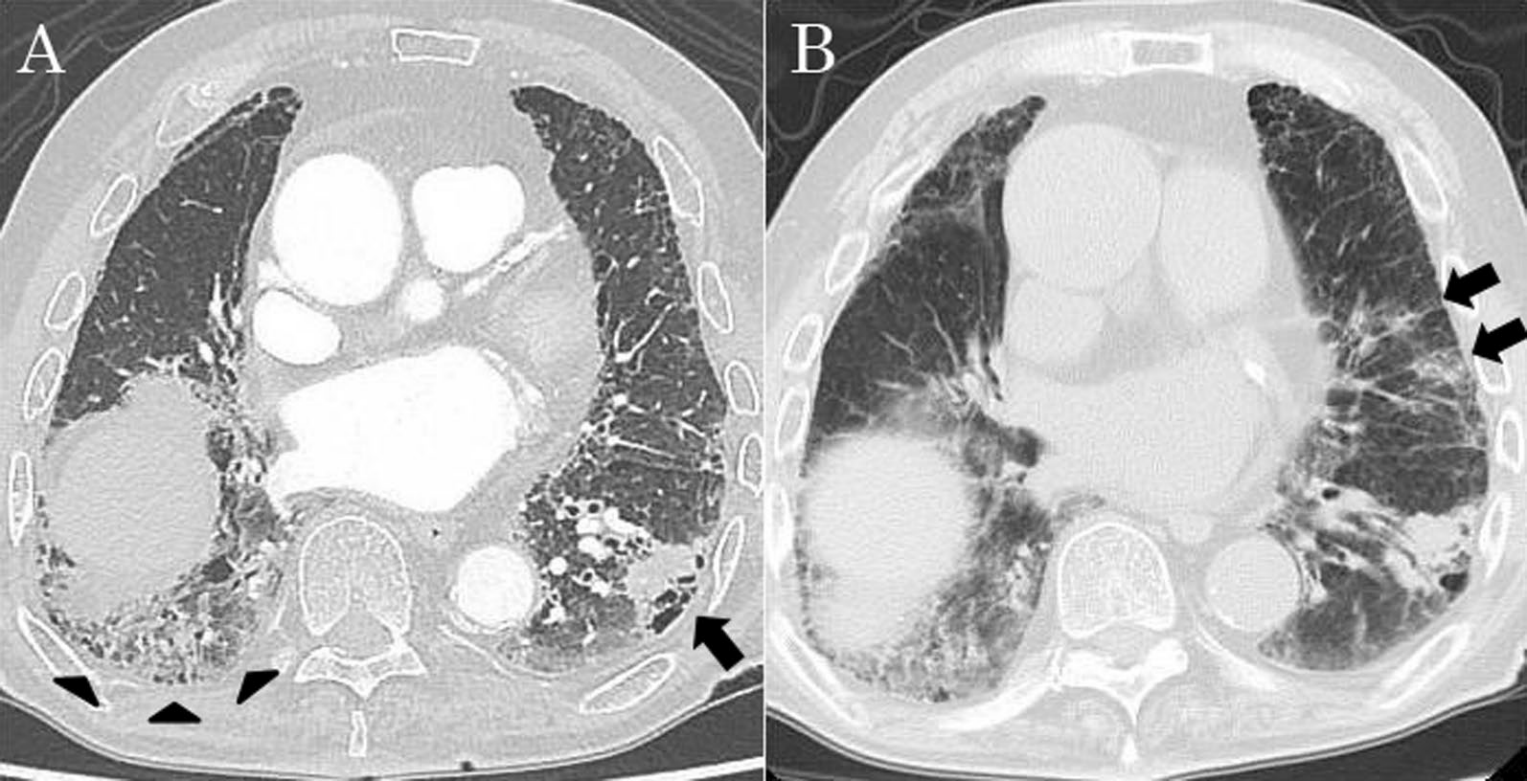
Chest computed tomography (CT) showing a 28-mm nodule (arrow) in the lower lobe of the left lung and usual interstitial pneumonia (arrowhead) in the lower lobes of bilateral lungs (**A**). When the patient contracted COVID-19, chest CT revealed ground-glass opacities (arrow) and showed that the tumor had enlarged to 35 mm in diameter (**B**)

**Fig. 2:**
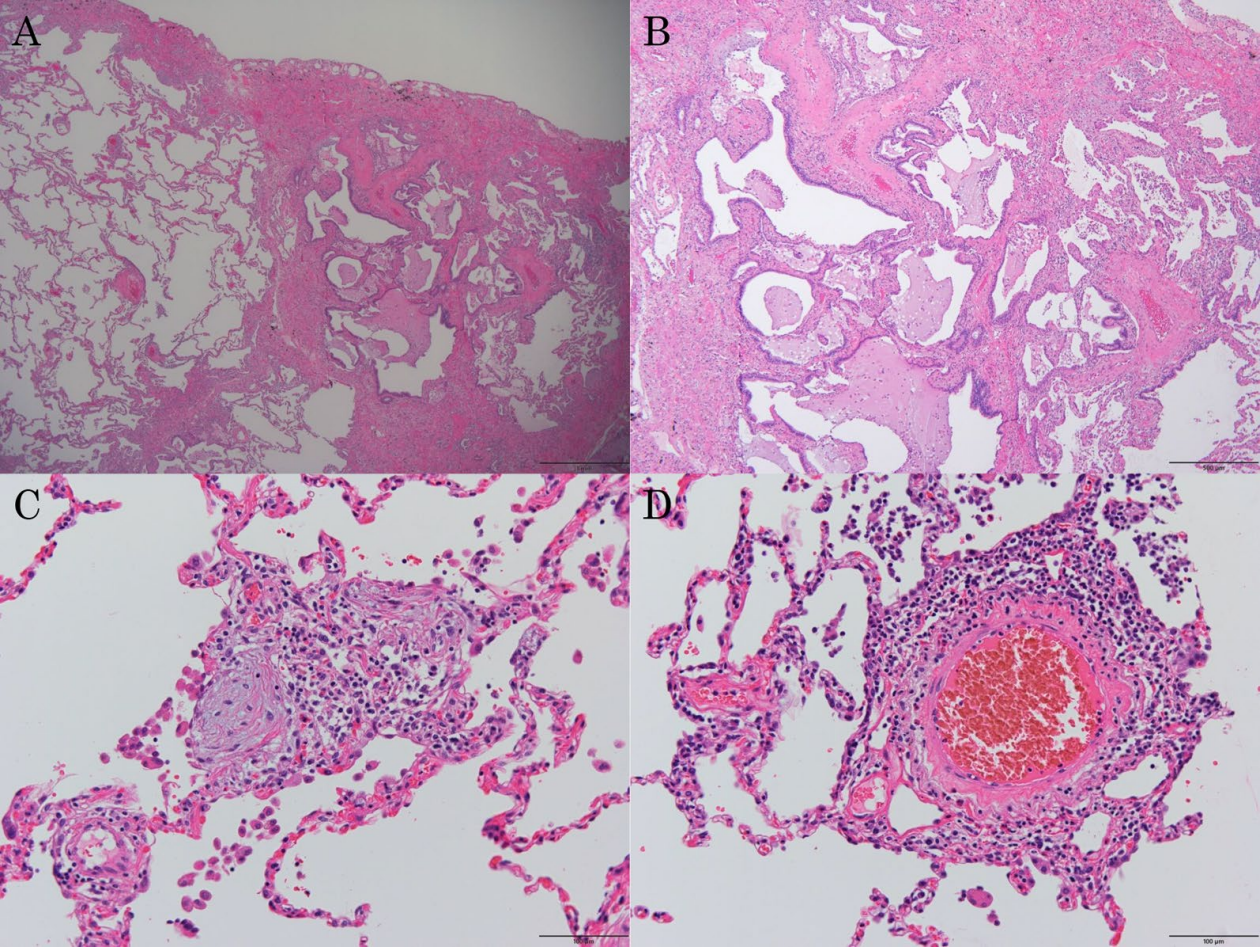
Pathological examination of the resected specimen showing usual interstitial pneumonia in the non-cancerous areas of the lung (**A**, **B**). Infiltration of immature fibroblasts is visible in the alveoli (**C**), and perivascular infiltration of inflammatory cells was observed (**D**)

An 80-year-old man was referred to our hospital because of a tumor in his left lung that was discovered on chest radiographs. Chest computed tomography (CT) showed a 28-mm nodule (Fig. 1A, arrow) in the lower lobe of the left lung and usual interstitial pneumonia (Fig. 1A, arrowhead) in the lower lobes of bilateral lungs. Fluorodeoxyglucose-positron emission tomography showed high accumulation in the nodule (maximum standard uptake value: 11.1). There was no accumulation in the hilar and mediastinal lymph nodes. Bronchoscopic examination was performed, which diagnosed squamous cell carcinoma. Pulmonary function testing revealed restrictive ventilatory impairment (Table 1). Using the modified GAP model for East-Asian populations with idiopathic pulmonary fibrosis (5), the stage was II. We planned to perform basal segmentectomy of the left lung. Predicted postoperative % vital capacity (%VC) and % diffusing capacity for carbon monoxide (%DLco) after basal segmentectomy were 59.9% and 62.4%. However, before the planned surgery, the patient contracted symptomatic COVID-19. Chest CT revealed ground-glass opacities (Fig. 1B, arrow) owing to COVID-19 and showed that the tumor had enlarged to 35 mm in diameter (Fig. 1B). The patient was admitted for surgery 7 weeks after COVID-19 infection. Viral antigen and polymerase chain reaction testing of pharyngeal swabs were negative at admission. Chest CT revealed that the ground-glass opacities decreased and there was no lymphadenopathy. Preoperatively, pulmonary function testing was repeated, which revealed decreased %VC and %DLco compared to those before COVID-19 infection (Table 1). The stage was III with the use of the modified GAP model (5). The 6-min walk test indicated a distance of 500 m, and the percutaneous oxygen saturation at the end of the test was 94%. We performed basal segmentectomy of the left lung by video-assisted thoracoscopic surgery. The operating time was 167 min, and the blood loss volume was 75 ml. The patient’s postoperative course was favorable, and he was discharged without the need for oxygen inhalational therapy 12 days after the surgery. Pathological examination of the resected specimen revealed squamous cell carcinoma and no metastases to the lymph nodes. The pathological stage was 1B. Usual interstitial pneumonia was revealed in the non-cancerous areas of the lungs (Fig. 2A, B). Additionally, the infiltration of immature fibroblasts in the alveoli (Fig. 2C) and perivascular infiltration of inflammatory cells (Fig. 2D) were observed, which were consistent with fibrotic change after inflammation owing to COVID-19 as previously reported (6). Three months after the surgery, the patient was alive without recurrence or acute exacerbation of the interstitial pneumonia. We did not administer any medicine for interstitial pneumonia, perioperatively. Pulmonary function testing 6 weeks after surgery revealed decreased %VC and %DLco compared to predicted value using the pulmonary function test before COVID-19 infection. Testing 12 weeks after surgery revealed persistently decreased %VC and improved %DLco (Table 1).

## Discussion

The spread of COVID-19 infection increased the number of patients treated for lung cancer after COVID-19 infection. There are reports of patients who underwent pulmonary resection for lung cancer after COVID-19 infection (1–4). The mortality risk with surgery after COVID-19 infection is increased for up to 5–6 weeks after COVID-19 infection (7). However, it is unclear how previous infection with COVID-19 affects perioperative complications in patients with interstitial pneumonia. In particular, patients with interstitial pneumonia require careful attention regarding acute exacerbation after surgery. Nakagomi et al. reported a patient who underwent pulmonary resection 1 month after COVID-19 infection (4). The patient died due to acute exacerbation of interstitial pneumonia on the 15th postoperative day (4). High mortality and acute exacerbation have been reported in patients with COVID-19 and interstitial pneumonia (8, 9). However, there are no data evaluating acute exacerbation of interstitial pneumonia after pulmonary resection in patients with previous infection with COVID-19. Regardless of COVID-19 status, a retrospective cohort study reported that the incidence of acute exacerbation of interstitial pneumonia after surgery was 9.3% (10). Sex (male), anatomical resection (lobectomy or segmentectomy), low %VC, and a usual interstitial pneumonia pattern on CT are associated with acute exacerbation of interstitial pneumonia (10), and the present patient had these risk factors. However, radiotherapy or chemotherapy could not be recommended in our patient owing to the severe interstitial pneumonia and his advanced age. Oxygen desaturation at the end of the 6-min walk test is a significant predictor of survival in patients with interstitial pneumonia (11). Our patient’s oxygen saturation was not abnormally low after the 6-min walk test. Therefore, we decided to perform pulmonary resection, and we expected a long survival time when the patient did well postoperatively without acute exacerbation.

Sakai et al. reported that organized fibrosis and inflammation might remain for a prolonged period after recovery from COVID-19, even with improved radiological findings (1). The present case had fibrotic change after COVID-19, pathologically. Although the pathological features were not specific for COVID-19, we recognized they were owing to COVID-19 considering his clinical course. Decreased %VC and %DLco after COVID-19 have been reported, and these pulmonary function parameters improved several months after infection (12). In contrast, patients undergoing delayed pulmonary resection (more than 8 weeks after diagnosis) had more pathological upstaging and decreased median survival in one study (13). If we wait for improvement of pulmonary function, patients may miss the surgical indication. We performed pulmonary resection 7 weeks after COVID-19 infection in our patient, in accordance with a report by the COVIDSurg Collaborative (7). There have been no reports of successful cases with severe interstitial pneumonia and restrictive ventilatory impairment after COVID-19 infection undergoing lung resection. Therefore, the changes in pulmonary function test results before and after COVID-19 infection and after surgery were very interesting findings in the present patient with severe interstitial pneumonia. In patients undergoing pulmonary resection for lung cancer immediately after COVID-19 infection, we should be prepared for prolonged decreased pulmonary function after surgery. The optimal timing of surgery after COVID-19 infection should be determined by considering the degree of lung cancer progression and pulmonary function, including restrictive ventilatory impairment owing to organized fibrosis and inflammation caused by COVID-19 infection. Studies involving a large number of patients are needed to evaluate acute exacerbation of interstitial pneumonia after pulmonary resection in patients with previous COVID-19 infection.

## Conclusions

We successfully performed basal segmentectomy of the left lung for lung cancer 7 weeks after COVID-19 infection in a patient with severe interstitial pneumonia and restrictive ventilatory impairment. Postoperative follow-up is required in patients with decreased pulmonary function.

## Data Availability

Applicable.

## Abbreviations

CT: Computed tomography
%VC: % Vital capacity
%DLco: % Diffusing capacity for carbon monoxide
